# Soil seed bank dynamics of two invasive alien plants in Nigeria: implications for ecosystem restoration

**DOI:** 10.1093/aobpla/plae003

**Published:** 2024-01-20

**Authors:** Gbenga F Akomolafe, Rusly Rosazlina, Bernard Omomoh

**Affiliations:** School of Biological Sciences, Universiti Sains Malaysia, 11800, Minden, Penang, Malaysia; Department of Plant Science and Biotechnology, Federal University of Lafia, PMB 146 Lafia, Nigeria; School of Biological Sciences, Universiti Sains Malaysia, 11800, Minden, Penang, Malaysia; Department of Forestry & Wood Technology, Federal University of Technology, Akure, Nigeria

**Keywords:** Invasive plants, restoration, seed banks, seed viability, vegetation

## Abstract

The assessment of seed banks could provide useful hints towards ensuring restoration planning and invasive species management. In this study, the impacts of two invaders such as *Hyptis suaveolens* and *Urena lobata* on the soil seed banks were investigated. We also assessed the seed characteristics of the invaders at the invaded sites. This was achieved using 10 sites each for *H. suaveolens-* and *U. lobata-*invaded habitats and -non-invaded habitats making a total of 30 sites. We collected 200 soil samples from each habitat type. A seedling emergence method was used to determine the seed bank recruitment of both invasive plants. The diversity indices of the above-ground vegetation of sites invaded by the two plants were significantly lower than those of the non-invaded sites. Only two plant species emerged from the seed banks of *H. suaveolens* and five plants from those of *U. lobata* when compared with non-invaded sites where 53 species emerged. A larger portion of the seeds was located in the soil’s lower layer at all the sites invaded by *H. suaveolens* while those of *U. lobata* and non-invaded sites were found in the upper layers and there are significant associations between the habitats. The lower soil layers of the two species have the highest percentage of viable seeds. These results help us to understand more about the invasiveness of both species as related to their impacts on the seed banks and native vegetation. It also indicates that the native species that emerged from the invaded seed banks could be used for the restoration of the invaded habitats.

## Teaser

Our study assessed the seed banks of two stubborn invasive plants (*Hyptis suaveolens* and *Urena lobata* ), towards ensuring restoration planning and management of affected sites in Nigeria. We discovered that these plants have greatly affected their seed banks and the standing vegetations. However, native plants such as *Oryza barthii* , *Heterotis rotundifolia* and *Celosia argentea* which emerged from the seed banks of both plants could be used as restorers of the affected sites. These results help us to understand more about the invasiveness of both plant species as related to their impacts on the seed banks and native vegetation.

## Introduction

Soil seed banks are reservoirs of seeds in the soil that represent both past, present and future plant community assemblage of the ecosystem (Gioria and Osborne 2010). They have important roles to play in the restoration of invaded ecosystems and the management of endangered plant species across the world (Richardson and Kluge 2008). Soil seed banks are relevant in the ecological restoration of degraded ecosystems because the viable and intact seeds present in the soil can grow under suitable conditions to re-establish the plant cover of affected areas. This is very useful in restoration projects (Skoglund 1992). The limitations of using other approaches in ecosystem restoration have necessitated the use of soil seed banks which are less labour intensive with very little technical know-how (Fowler *et al*. 2015). This could involve the transfer of a removed topsoil containing potentially viable seeds from a natural area to a degraded area for rapid restoration of natural vegetation (Koch 2007). The use of soil seed banks for the restoration of degraded sites has been proven successful in the restoration of degraded mine sites (Parrotta and Knowles 2001), farmlands and roadsides (Vécrin and Muller 2003) across different parts of the world. The ability of plants to effectively compete with others in their native community is also dependent on their seed viability and seed bank density. A seed bank may either contain seeds that are viable in the soil for less than a year (transient) or seeds that remain viable in the soil for a long period (persistent) (Thompson and Grime 1979). The persistent seed bank is further classified as short-term persistent (seeds remain viable in the soil for 1–5 years) and long-term persistent seed banks (seeds remain viable in the soil for over 5 years) (Walck *et al*. 2005). This persistence of seed viability is dependent on the prevailing environmental factors and the species’ inherent traits (Louda 1989).

The invasiveness of alien plants can be established by examining their soil seed banks (Gioria *et al*. 2014). The seeds of invasive plants are known to survive better than their native counterparts in the soil (Masaki *et al*. 1998). This is because a lot of native plants with short-lived seeds usually have difficulty in germination once the seeds enter the seed bank in the soil (Chambers and MacMahon 1994). Besides, other factors such as the activities of soil fauna may also hinder the survival of those seeds in the soil. Therefore, the number of viable, non-viable, intact and physically damaged seeds in the soil seed bank demonstrates the relationship between the input of seeds into the soil and their degradation (Moravcová *et al*. 2007). It also indicates the type of seed bank.

Considering the relevance of seed banks in the restoration of ecosystems, only a few studies have assessed the relationships between plant invasions and their corresponding soil seed banks in tropical Africa (Gioria *et al*. 2014). To have adequate information for managing invasive plants and recognizing their impacts on the invaded communities, there is a need to compare the above-ground vegetation and soil seed banks of invaded and non-invaded sites (Manchester and Bullock 2000; Hejda *et al*. 2009). It is also important to assess the seed bank composition to enable the detection of invasive and rare species hidden in the soil as buried seeds (Schneider and Allen 2012). The changes arising from the introduction of invasive plants usually affect the soil seed banks of native plants (Gioria and Osborne 2010). Therefore, to have an improved restoration plan and management of invasive plants, understanding the roles of seed banks in vegetation dynamics of invaded and non-invaded habitats is needed (Grewell *et al*. 2019).

*Hyptis suaveolens* of the family Lamiaceae is an annual herbaceous plant that originated from the Neotropical regions and has been introduced into the tropical and sub-tropical regions of the world. Over the years, this plant has been naturalized in its introduced ranges due to its adaptability to climatic conditions (Padalia *et al*. 2015). *H. suaveolens* can invade disturbed habitats with the massive production of sticky seeds (Sharma *et al*. 2007). It can persist in invaded ecosystems due to its propagule banks and ability to remain dormant during unfavourable environmental conditions (Parsons *et al*. 2001). Its invasiveness is expressed in its ability to displace native plant species with the formation of dense colonies. Several countries including the Philippines, Singapore, Hawaii, India, Australia, Nigeria (David *et al*. 2021) and so on have reported the incidence and severity of its invasion. Some considered it as an environmental weed, noxious weed and serious invader. This is due to the damage it causes to farmlands by competing with crops and reducing their yields. Its preference for habitats includes roadsides, farmlands, rangelands, open woodlands, grasslands, wetlands, wastelands etc. (Sharma *et al*. 2007). In Nigeria, *H. suaveolens* has been predicted to have the potential to spread to several parts of the country with different ecological attributes (David *et al*. 2021). One of the advantages of *H. suaveolens* over the natives is its fast-growing nature and the ability of the seeds to germinate under diverse temperature ranges (Sharma *et al*. 2009). Besides being an invader, *H. suaveolens* has also been regarded as economically useful due to its medicinal value (Wiersema and León 1999; Towns and van Andel 2014).

*Urena lobata* of the family Malvaceae has also been regarded as a noxious weed in many parts of the world (Akomolafe and Nkemdy 2020) and has been recorded in the global compendium of weeds. It can grow very fast and spread speedily across diverse habitats by propagating through the seeds (Langeland *et al.* 2008). This makes it easily outcompete and dominate native plant communities within a short period. Its seeds usually get stuck to the animals which act as agents of dispersal (Austin 1999). *U. lobata* is an erect, woody, shrub-like and perennial plant that usually grows up to 3 m (Francis 2000). *U. lobata* has become naturalized and occupied many landscapes in many African countries, particularly Nigeria after its introduction. In Nigeria, it has been found to grow widely in both rainforest and savanna areas (Akomolafe and Nkemdy 2020; Njoku *et al*. 2021). As a highly tolerant plant, *U. lobata* has been found growing in different soil types and habitats such as pastures, wastelands, wetlands, roadsides and farmlands (Francis 2004). As an aggressive invader, it can also overpopulate native communities and alter the community structure and functions (Austin 1999).

Despite the impacts of these two plants on the invaded ecosystems in Nigeria, not many studies have focused on the ecology of their seed bank which is mostly the long-lived life stage that influences the restoration and succession of invaded ecosystems (Grewell *et al*. 2019). Hence, the objectives of this study include: (i) to assess the seed characteristics of these two invaders which include their numbers, distribution in the soil profile (lower and upper layers) and how intact and viable they are in the soil, as these are important contributors to their persistence and invasion; (ii) to investigate the seedling emergence of the soil seed banks of sites invaded by the two species and uninvaded ones; (iii) to compare the seed bank composition with the above-ground plant community at both invaded and non-invaded habitats. The two species were chosen because of their prominence in several plant communities at different landscapes in Nasarawa State, Nigeria.

## Methods

### Study area

**Figure 1.**
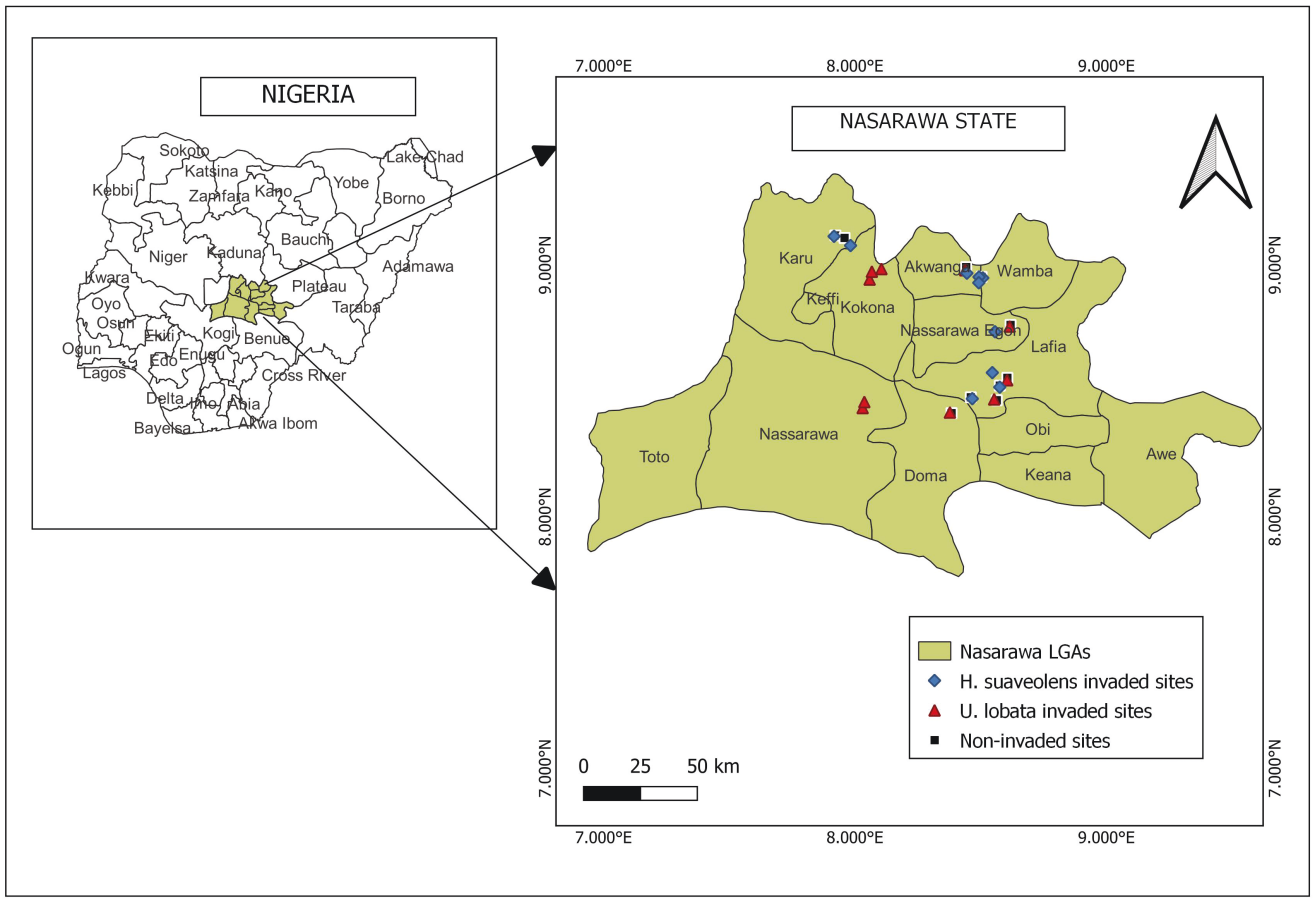
Study area map of Nasarawa State, Nigeria showing the invaded and non-invaded habitats.

This study was conducted in Nasarawa State, North Central Nigeria (Fig. 1). Nasarawa State with an area of 27 117 km^2^ is predominated by guinea savanna vegetation comprising mainly grasses, woody shrubs and very few trees. Nasarawa State has 13 local government areas with Lafia as its capital city. The people of the State are generally involved in the farming of groundnut, sesame, soybeans, millet, maize and yam (Kwon-Ndung *et al*. 2016).

### Study site/sampling procedure

**Figure 2.**
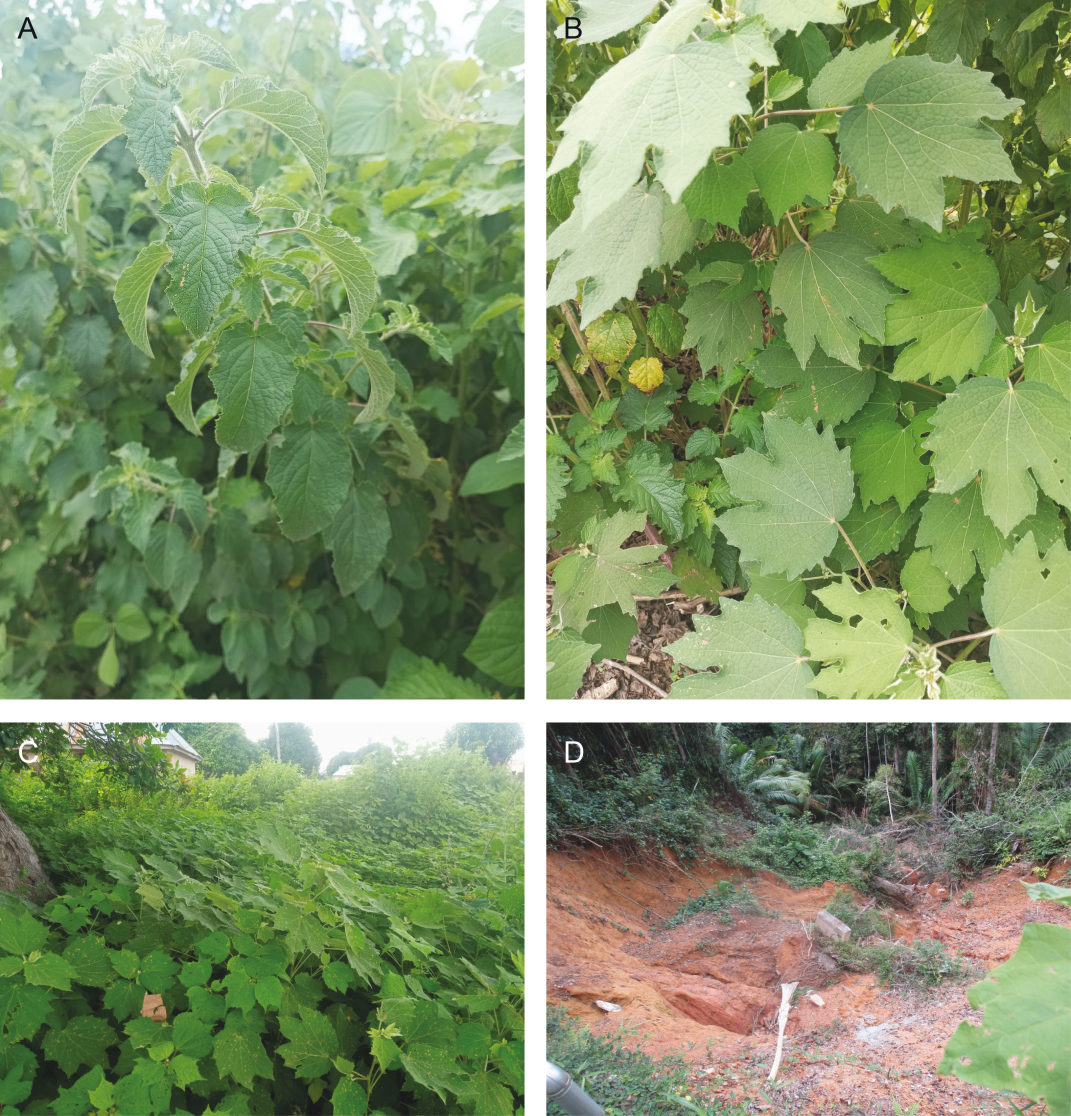
(A) *Hyptis suaveolens*. (B) *Urena lobata* plant. (C) Invaded site. (D) Non-invaded site.

Invaded habitats of *H. suaveolens* (HS-invaded sites, Fig. 2A) and *U. lobata* (UL-invaded sites, Fig. 2B) were chosen throughout Nasarawa State, Nigeria, depending on their distribution (Fig. 1, Table 1). Other ecological factors were taken into consideration in the selection of the habitats. We ensured that the habitats have similar land use types, human disturbance and structure. For example, all the selected habitats were chosen at abandoned/open land, low elevation range and herbaceous/shrub-like vegetation structure. Within this State, we sampled three habitat types which include 10 HS*-*invaded sites, 10 UL*-*invaded sites (Fig. 2C) and 10 non-invaded sites (Fig. 2D). Stands with a considerable number of individuals of the mature plants, also with not less than 60% plant cover were chosen for the seed bank sampling study. All the sites chosen as invaded have reached an advanced stage of invasion whereby both plants have completely dominated the sites thereby forming monospecific colonies with very minimal presence of natives. Voucher plant specimens collected from the study sites were air-dried, pressed and deposited at the Herbarium of the Department of Plant Science, Federal University of Lafia, Nigeria.

**Table 1.** Number and percentage of total, damaged, undamaged, viable and unviable seeds in different soil layers of the two species, *H. suaveolens* and *U. lobata* and non-invaded habitat types.

Seed Parameter	HS-invaded site			UL-invaded site			Non-invaded site		
	Upper layer	Lower layer	Total	Upper layer	Lower layer	Total	Upper layer	Lower layer	Total
Total number of seeds	322	645	967	543	344	887	2555	1310	3865
Percentage of total	33.3	66.7	100	61.2	38.8	100	66.11	33.89	100
Number of damaged seeds	87	107	194	141	40	181	505	198	703
Percentage of damaged seeds	8.99	11.07	20.06	15.89	4.51	20.39	13.07	5.12	18.19
Number of undamaged seeds	235	538	773	402	304	706	2160	1002	3162
Percentage of undamaged seeds	24.30	55.64	79.94	45.32	34.27	79.59	55.89	25.93	81.82
Number of viable seeds	269	512	781	317	268	585	2018	968	2986
Percentage of viable seeds	27.82	52.95	80.77	35.74	30.21	65.95	52.21	25.05	77.26
Number of unviable seeds	53	133	186	226	76	302	586	293	879
Percentage of unviable seeds	5.48	13.75	19.23	25.48	8.57	34.05	15.16	7.58	22.74

The soil samples for analysing the seed bank were collected at the end of the growing season between November 2021 and January 2022. This is the period when the plants have completed fruiting and most of them have fallen to the ground. Plots of size 2 m × 2 m were used as sampling plots. At each site, soil samples were collected using soil cores made of steel frames (dimension: height = 10 cm, size = 10 × 10 cm^2^) (Petrulaitis *et al*. 2022). At each site, 10 plots were established for the soil collection. Within each plot, two soil cores were taken making a total of 20 soil samples per site and 200 per habitat type Out of these 600 soil samples (invaded and non-invaded habitats inclusive), 300 samples (100 per each habitat type) were divided into two sets of subsamples inside each core: 0–5 cm (upper layer) and 5–10 cm (lower layer), making a total of 600 subsamples. These 600 subsamples were to be used for the seed damage analysis while the remaining 300 samples were used for the seedling emergence study. All the samples were properly labelled and packaged on the field before being transported to the laboratory. Thereafter, the soil samples were air-dried at room temperature by opening the containers. The debris and other organic matter in the soil samples were carefully removed.

### Seed damage analysis

The 600 dried soil subsamples (invaded and non-invaded sites inclusive) were hand-crushed, and the seeds were separated using different sets of sieves of sizes 3, 2, 1 and 0.25 mm (Petrulaitis *et al*. 2022). The seeds of *H. suaveolens* and *U. lobata* were collected from each subsample using pincers and separated into different containers. As for the non-invaded sites, all the seeds were categorized as one type regardless of the plant species. The dissecting microscope was then used to identify and separate damaged seeds from undamaged ones. Damaged seeds are the ones with visible breakage of the seed coat. The numbers of both were counted in each subsample. This is important to understand the regenerative ability of the soil seed bank.

### Seed viability test

This was conducted on all the undamaged seeds obtained from the soil seed bank. The viability test was done using TTC test which has been recognized as a standard staining method (Baskin and Baskin 1998; Petrulaitis *et al*. 2022). The mechanical scarification method of opening the micropyle of each seed was done by scratching the seeds on a hard surface to enable the embryo to be easily penetrated by the TTC compound. After the seeds had been treated with the TTC, they were removed from the TTC and allowed to stay for almost 24 hours in the dark in a growth chamber. The seeds were sectioned and observed under the microscope to view the colour of the embryos. Those embryos with carmine colouration were regarded as viable whereas those with white or pink colouration were noted as non-viable (Starfinger and Karrer 2016).

### Seedling emergence assay

This has been described as the most accurate method of determining soil seed viability and comparing seedling recruitment with above-ground vegetation (Nicol *et al*. 2003). The 300 soil samples (200 samples for invaded habitats and 100 samples for non-invaded habitats) that were kept for the seedling emergence assay were air-dried. Stones and gravel were removed by passing the soil samples through a sieve of 4 mm mesh size. The soil was put in perforated 43 cm diameter plastic bowls and placed in a screen house at an average temperature of 39 °C. The soils were watered daily and germinated seedlings were counted, identified and removed at the end of the experiment. This experiment was monitored for five months. The identification of the plants was done using taxonomic floras and confirmed using the online database of the International Plant Name Index. Those plants whose identities are difficult to establish at the end of the five months were transplanted and grown to maturity to enable easy identification.

### Vegetation impact analysis

Vegetation sampling was carried out during the wet season (October–December 2021) when the plant community reached maximum productivity. The same number and size of sampling plots (i.e. 10 plots of size 2 m × 2 m) established earlier in each of the sites of the invaded and non-invaded habitats were used to determine the ecological impact of the invasive plant species on the invaded plant community. A total of 200 plots were randomly placed at the invaded habitats of both plants while 100 plots were used at the non-invaded habitat. All rooted plant species were identified and counted. Randomization was done using the lucky draw method by drawing out numbers to generate points where quadrants were laid. Voucher specimens of the species that could not be identified in the field were collected and identified in the herbarium of the Department of Plant Science and Biotechnology, Federal University of Lafia. To assess the impact of the invasive plants on their communities, the following diversity indices were estimated: species richness, evenness index, Shannon index and Simpson index. These diversity indices were also calculated for the non-invaded sites and compared with invaded ones.

### Data analyses

We analysed the seed density by counting the number of seeds per subsample per study site. This gives us the mean seed density per unit square metre (Petrulaitis *et al*. 2022). We tested the normality of the seed bank data and discovered the data were skewed to the left (not normally distributed). Therefore, the equivalent non-parametric analyses were employed. For instance, Kruskal–Wallis (an equivalent of one-way ANOVA) was used to assess the differences between the sample medians. Also, the Chi-square (*χ*^2^) test was used to test the association between seed damage proportions (damaged and undamaged) and soil layers of the two species. The Chi-square and other descriptive statistics were carried out using the Paleontological Statistics (PAST) 3.0 package. The species composition of the soil seed bank was compared with the corresponding above-ground vegetation for both invaded and non-invades sites using Sorensen’s index of similarity with the formula:


$$\begin{array}{l} SIS=\frac{C}{A+B}\;X\;100 \end{array}$$

where *C* is the number of species common to both seed banks and above-ground vegetation, *A* is the number of species in the seed bank and *B* is the number of species in above-ground vegetation. A one-way analysis of similarities (ANOSIM) was used to test the similarity of species composition between: (i) invaded and non-invaded aboveground plant communities and (ii) invaded and non-invaded seed banks. This was tested using the Euclidean distance between each pair of parameters as the similarity index. A high *R*-value indicates the dissimilarity between each pair. The Student’s *t*-test was used to compare the means of the total species richness (number of species per site) between the invaded and non-invaded sites of the above-ground vegetation as well as those of the invaded and non-invaded soil seed banks. The analysis was done using the PAST version 3.17 software.

## Results

### Seed distribution and density across soil layers

The total number of damaged and undamaged seeds collected from the soil samples was 967 seeds at *H. suaveolens*-invaded habitat and 887 seeds at the *U. lobata-*invaded habitat. At the non-invaded habitat, 2555 seeds were found at the upper layer while 1310 were found at the lower layer, thereby making a total of 3865 seeds. (Table 1). A larger percentage (66.7%) of the seeds were located at the lower layer (5–10 cm) at all the sites invaded by *H. suaveolens.* As for UL-invaded and non-invaded habitats, a larger portion (61.2%) of the seeds were found at the upper layers and there are significant differences between the sites (χ^2^ = 63.53, *P* < 0.0001). For instance, out of the total 967 seeds recorded at the *H. suaveolens* habitat, 645 were found at the lower layer while 322 were at the upper layer. Also, for *U. lobata,* out of the 887 seeds, 543 were found at the upper layer while 344 were at the lower layer. By implication, the lower soil layer of HS-invaded habitat has the highest percentage of viable seeds (52.95%), the upper soil layers of UL-invaded and non-invaded habitats have the highest percentage of viable seeds (35.74% and 52.21% respectively). There was a significant association (*χ*^2^ = 61.96, *P* < 0.0001) between the soil layer and seeds of the species at all the sites, with a higher proportion (11.07%) of damaged seeds of *H. suaveolens* found in the lower soil layer, compared to *U. lobata* and non-invaded habitat which had 15.89% and 13.07% of damaged seeds respectively in the upper soil layers.

**Figure 3.**
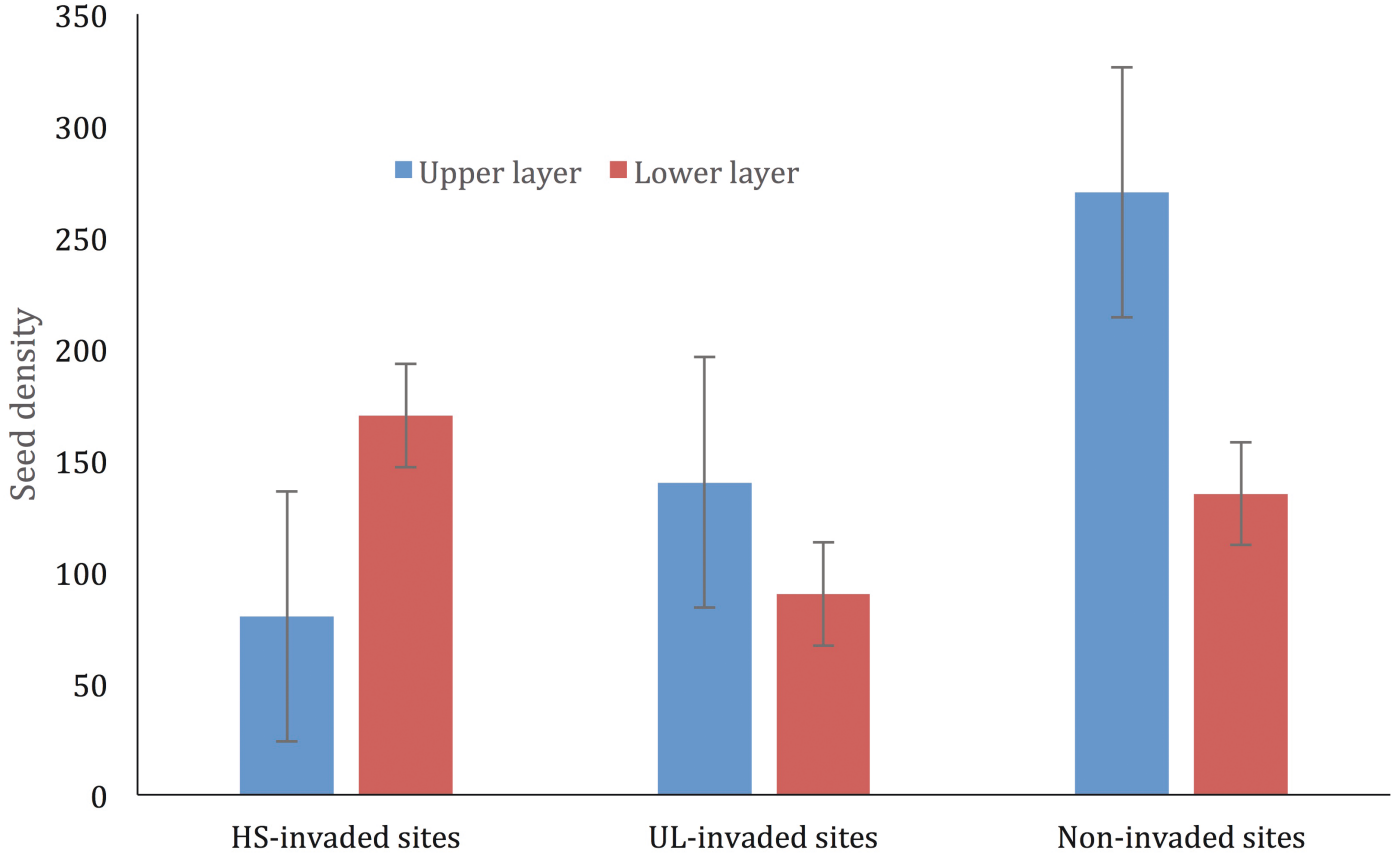
Mean number of seeds per square meter in the soil layers at the different habitat types.

The highest mean density of seeds at the lower layer was found in *H. suaveolens* invaded habitat whereas the highest mean density of seeds at the upper layer was found in non-invaded habitat (Fig. 3). Invariably, the lowest mean density of seeds at the lower soil layer was found in UL*-*invaded habitat while the lowest mean density at the upper layer was found in *H. suaveolens* invaded habitat. The differences in the mean density between the upper and lower layers of the sites were significant (*P* < 0.05).

### Seedling emergence/seed bank vegetation dynamics

**Figure 4.**
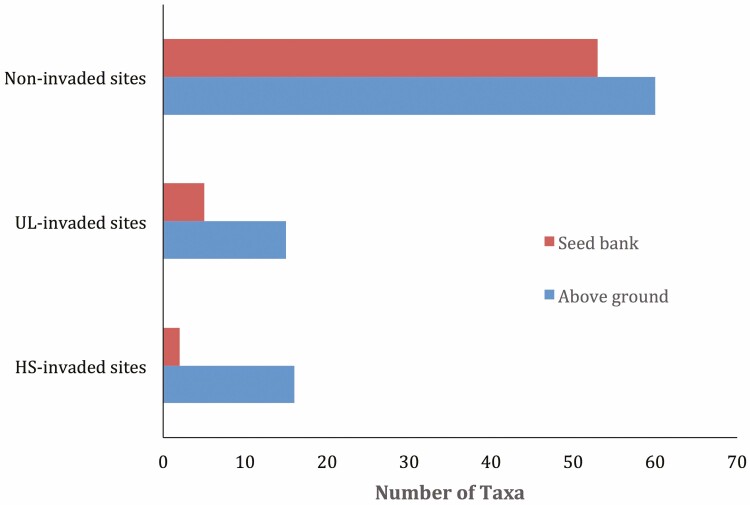
Comparison of the number of taxa at above-ground plant community and seed bank compositions of invaded and non-invaded habit.

Out of the 16 species found at the HS*-*invaded habitat above ground, only two (2) of them emerged from the corresponding seed banks (Fig. 4). The species include *H. suaveolens* and *Oryza barthii*. Out of these two species, only *Oryza barthii* is a native species in Nigeria. Out of the number of seedlings that emerged from the HS-invaded habitat, *O. barthii* had 45% occurrence. Also, for the UL*-*invaded habitat, only five species emerged from the seed bank out of the 15 species recorded at the corresponding above-ground vegetation. The species include *U. lobata, Tridax procumbens, Heterotis rotundifolia, Celosia argentea* and *Amarathus spinosus* with percentage occurrence of 40%, 10%, 15%, 13% and 22%, respectively. Out of these five species, only *Heterotis rotundifolia* and *Celosia argentea* are native to Nigeria. The Sorenson similarity in species composition between the above-ground and soil seed bank vegetations of *H. suaveolens* is smaller (11.1%) than that of *U. lobata* (25%) invaded habitat. However, 53 plant species emerged from the seed bank of non-invaded habitat out of the 60 species earlier recorded at the corresponding above-ground vegetation. This makes a Sorenson similarity index of 46.9%.

### Above-ground vegetation diversity

The total number of species identified at the above-ground vegetations of the HS-invaded habitat is 16 while that of *U. lobata* is 15 (Table 2). Similarly, the habitats of both species have very low diversity indices and are not significantly different from each other (see Table 2). However, the highest mean Shannon and evenness indices among the invaded habitats were observed in the UL-invaded habitat. As for the non-invaded habitat, a total of 60 species with 4523 individuals were observed. These non-invaded habitats also recorded the highest diversity indices which are significantly different (*P* < 0.05) from those of invaded habitats of both plants. Only one species, *Daniellia oliveri* was found to be common to the invaded and non-invaded habitats (see Supporting Information—Table S1). However, three species were common to both *H. suaveolens* and *U. lobata* invaded habitats. They include *Alchornea cordifolia, Eragrostis ciliaris* and *Daniellia oliveri.*

**Table 2.** Diversity indices/community characteristics of above-ground plant community of sites invaded by *H. suaveolens* and *U. lobata* and non-invaded ones.

Community characteristics	HS-invaded sites	UL-invaded sites	Non-invaded sites
Taxa_S	16 ± 0.58	15 ± 0.06	60 ± 1.53
Number of individuals	2053 ± 1.15	1964 ± 1.42	4523 ± 2.62
Simpson index	0.88 ± 0.03	0.94 ± 0.02	0.35 ± 0.04
Shannon index	0.67 ± 0.02	0.85 ± 0.03	4.57 ± 0.02
Evenness index	0.56 ± 0.04	0.79 ± 0.02	2.43 ± 0.03

Values represent mean ± standard deviation.

### Impact of the invasive plants on the resident communities

The species richness was discovered to be significantly different between the invaded vegetation and non-invaded ones (*t* = 43.2, *P* < 0.0001). Likewise, a significant difference was observed between the species richness of the invaded seed bank and non-invaded ones (= 47.98, *P* < 0.0001). One-way ANOSIM between the above-ground vegetation of invaded sites and the above-ground vegetation of non-invaded sites revealed a very low similarity in species composition (*R* = 0.99, *P* = 0.34). The same high level of dissimilarity was also observed in the species composition between invaded and non-invaded seed bank vegetation’s (*R* = 0.89, *P* = 0.33).

## Discussion

The results showed that a larger portion of the seeds was located in the lower sub-samples of the soil than in the upper layer for *H. suaveolens* as compared with the *U. lobata* and non-invaded habitats which had theirs in the upper soil layer. This has already been reported as typical of many plant species (Berge and Hestmark 1997; Moravcová *et al*. 2007; Gioria *et al*. 2019; Petrulaitis *et al*. 2022). This distribution of seeds across the soil profile was said to have been influenced by some biological features such as the size and shapes of the seeds (Bekker *et al*. 1998; Pérez-Fernández *et al*. 2002). For instance, the small seed size of *H. suaveolens* usually facilitates the burial of the seeds deep in the soil due to the ease of penetration into small openings or cracks in the soil. This reduction in the seed size coupled with the production of a large number of seeds enabled the plant to avoid predators, hence promoting its invasion success (Raizada 2006).

On the contrary, the larger portion of the seeds of *U. lobata* at the upper soil layer could be due to the morphology of the seeds. The seeds of *U. lobata* are kidney-shaped which is different from a spherical shape. It has been reported that seeds with spherical or round shapes usually penetrate deeper into the soil as a result of a reduction in friction (Pérez-Fernández *et al*. 2002). Besides, environmental factors including the nature of the habitat and activities of micro and macrofauna in the soil do determine how seeds are distributed down the soil profile (Chambers and MacMahon 1994; Vander Wall 2003). Also, the presence of a higher percentage of viable seeds in the lower layer of the soils of *H. suaveolens* invaded habitats could be because the seeds were kept deep in the soil far away from mechanical destruction which could affect their viability. The microclimatic conditions in the lower part of the soil could be conducive enough to keep the seeds viable (Raizada 2006). The longevity of these viable seeds in the soil was not tested in this study. However, *H. suaveolens* is known to form a persistent propagule bank in the soil which aids its invasion (Sharma *et al*. 2007).

The low density of seed banks exhibited by the two species in the soil layers at the invaded habitats are contradictory to the regular pattern since invasive plants are known to exhibit high fruit production per individual plant (Thompson and Pellmyr 1989). *H. suaveolens* flowers are known to be pollinated by numerous pollinators thereby leading to the production of large numbers of fruits and seeds (Raizada 2006), hence adding more seeds to the seed bank. *H. suaveolens* as a prolific producer of seeds has been reported to always produce up to a range of 2000 to 3000 seeds per m^2^. However, in this study, the highest number of seeds of *H. suaveolens* per m^2^ is 161. This low density of seeds of the plant in this study could be attributed to the habitat conditions and stand characteristics (Petrulaitis *et al*. 2022). On the contrary, the non-invaded habitat was observed to have the highest seed density in this study.

The ability of plant species to germinate from soils after ecosystem disturbances is enhanced by the soil seed banks (Cui *et al*. 2013). The recruitment of native plant species from the seed bank is fundamental to the recovery and restoration of disturbed ecosystems. However, the emergence of invasive plants from the seed banks usually drives the ecosystems towards secondary succession/invasion, hence preventing its restoration (Grewell *et al*. 2019). In this study, the invaded habitats of both *H. suaveolens* and *U. lobata* demonstrated low seed banks. This is because only 12.5% and 33.3% of the plants present in the aboveground vegetation emerged from the corresponding seed banks respectively whereas in the non-invaded habitat, 88.33% of the aboveground vegetation emerged from the seed bank. This also indicates the negative impact of the invasive plants on their seed banks. The low seed banks observed at the invaded sites could have been caused by the reduction in seed germination due to allelopathy, resource competition or changes in soil chemistry by the invasive plants (Gooden and French 2014; Ruwanza, 2016). Indeed, several studies have reported both *H. suaveolens* and *U. lobata* to exhibit allelopathic effects on other neighbouring plants in their invaded habitats (Islam and Kato-Noguchi 2013; Poornima *et al*. 2015; Kaliyadasa and Jayasinghe 2018).

There is hope for the restoration of UL*-*invaded sites due to the presence of more native plant species in the seed bank composition. This means that the *U. Lobata* invaded sites have not reached a stage of secondary invasion due to the absence/dominance of exotic species in the seed bank (Ruwanza *et al*. 2013; Gioria *et al*. 2014). Comparing the above-ground vegetation and soil seed bank composition of invaded and non-invaded sites will help in identifying persistent native species that could be used for the restoration of invaded ecosystems (Manchester and Bullock 2000; Hejda *et al*. 2009). By implication, the result of this study is very useful to managers of invaded habitats because the native species that were recruited from the seed banks of *U. lobata* invaded sites can be propagated massively to prevent secondary invasion, and hence restore the invaded ecosystem. *Oryza bathii, Heterotis rotundifolia* and *Celosia argentea* which are the native species recruited from the invaded habitats could be used to serve the purpose of restoration of *H. suaveolens* and *U. lobata* invaded habitats in Nigeria. Also, it is worth noting that the seeds of both *H. suaveolens* and *U. lobata* were able to be recruited from their respective seed banks. This further demonstrated their persistent invasiveness.

The low similarity in the species compositions between the above-ground and seed bank vegetations for the two plant species indicated that there may be a change in the plant species compositions of the invaded habitats in response to ecosystem disturbances in the future (Wisheu and Keddy 1991). The reverse was the case for uninvaded ones where there was a slightly higher similarity between the above-ground and seed bank compositions. This result also agrees with other studies on the low species composition similarities between above-ground and seed bank vegetations of habitats invaded by some plants (Hopfensperger 2007; Grewell *et al*. 2019).

This is a piece of useful information on the seed bank responses of these invaders to restore their invaded ecosystems. This is because the seed bank shows the regenerative ability of an ecosystem and its plants’ recruitment from the soil after the removal of the above-ground vegetation (Grewell *et al*. 2019). An invader can only be controlled successfully in an ecosystem if such an ecosystem can regenerate the non-target native plants from the soil after the removal of the above-ground vegetation together with the invader. This also means that the invader cannot regenerate from the seed bank (Alexander and D’Antonio 2003). Therefore, the responses of seed banks and above-ground vegetation to control measures should be prioritized in any sustainable invasive plant management strategy (Grewell *et al*. 2019). Also, due to the formation of persistent seed banks by *H. suaveolens* and *U. lobata* as observed in this study, the removal of their above-ground biomass before the production of seeds could be an effective management method. This will ensure that it is only the non-target native species that will emerge from the seed bank, hence restoring the invaded habitats.

Since the seed bank is a potential source of propagation for the regeneration of degraded ecosystems, understanding the dynamics of the seed bank is an important key to restoring systems. Biological invasion is one of the major threats to the conservation and restoration of degraded ecosystems, which is why this study is so important and has shown the effects of invasion both on the above-ground vegetation and on the seed bank that ensures the maintenance of plant communities in a future time.

## Conclusion

This study has provided more information on the invasiveness of both *U. lobata* and *H. suaveolens* in relation to their impacts on the corresponding soil seed banks and native vegetation in Nigeria. The seed characteristics of both plants could have contributed significantly to their invasion success. The inability of more native species to emerge from the soil seed banks of sites invaded by both species is a signal of the extent of the impacts of the invaders in preventing natural restoration of the sites. The identified native species including *O. barthii, H. rotundifolia* and *C. argentea* which emerged from the seed banks of both plants could be used as restorers of the invaded habitats either through mass propagation or by allowing them to emerge from the seed banks naturally. This study calls for urgent and effective measures to be implemented for the control and/or management of these invasive plants to ensure the restoration of the affected habitats.

## Supporting Information

The following additional information is available in the online version of this article –

Table S1: Checklist of plants identified at both invaded and non-invaded above-ground vegetations.

plae003_suppl_Supplementary_Tables_S1

## Data Availability

The datasets generated during and/or analysed during the current study are available from datadryad.org: https://doi.org/10.5061/dryad.cvdncjt93.
