# Supplementary material for: Soil seed bank dynamics of two invasive alien plants in Nigeria: implications for ecosystem restoration
Source: AoB Plants. 2024 Jan 20;16(2):plae003. doi: 10.1093/aobpla/plae003 (PMC10880880; doi:10.1093/aobpla/plae003)
Supplement: plae003_suppl_Supplementary_Tables_S1 [file plae003_suppl_supplementary_tables_s1.pdf]

SUPPLEMENTARY MATERIAL

Table 1: Checklist of plants identified at both invaded and non-invaded above-ground vegetations

S/N	Species	Family	Non-invaded sites	*H. suaveolens*	*U. lobata*
1	*Ageratum conyzoides*	Asteraceae	+	+	-
2	*Alchornea cordifolia*	Euphorbiaceae	-	+	+
3	*Amarathus spinosus*	Amaranthaceae	+	-	+
4	*Andropogon tectorum*	Poaceae	+	-	-
5	*Anogeissus leiocarpa*	Combretaceae	+	+	-
6	*Aspilia africana*	Asteraceae	+	-	-
7	*Brachiaria alata*	Poaceae	+	-	-
8	*Brachiaria jubata*	Poaceae	-	+	-
9	*Calopogonium mucunoides*	Fabaceae	-	-	+
10	*Calotropis procera*	Apocynaceae	+	-	+
11	*Celosia argentea*	Amaranthaceae	-	-	+
12	*Cenchrus biflorus*	Poaceae	+	-	-
13	*Chloris pilosa*	Poaceae	+	+	-
14	*Chromolaena odorata*	Asteraceae	+	-	+
15	*Cissampelos mucronata*	Menispermaceae	-	-	+
16	*Combretum mollis*	Combretaceae	+	+	-
17	*Combretum platypterum*	Combretaceae	+	-	-
18	*Commelina benghalensis*	Commelinaceae	+	-	-
19	*Dactyloctenium aegyptium*	Poaceae	+	+	-
20	*Daniellia oliveri*	Fabaceae	+	+	+
21	*Datura stramonium*	Solanaceae	-	-	+
22	*Desmodium velutinum*	Fabaceae	+	-	-

23	*Elytrophorus spicatus*	Poaceae	-	+	-
24	*Emilia praetermissa*	Asteraceae	+	-	-
25	*Erythrophleum suaveolens*	Fabaceae	+	-	-
26	*Eragrostis ciliaris*	Poaceae	-	+	+
27	*Eriosema grisum*	Fabaceae	+	-	-
28	*Euphorbia hirta*	Euphorbiaceae	+	-	+
29	*Euphorbia poissonii*	Euphorbiaceae	+	-	-
30	*Gomphrena celosioides*	Amaranthaceae	+	+	-
31	*Grewia mollis*	Malvaceae	+	-	-
32	*Heteropogon contortus*	Poaceae	+	-	-
33	*Heterotis rotundifolia*	Melastomatacea e	-	-	+
34	*Hyperrhenia rufa*	Poaceae	+	-	-
35	*Hyptis suaveolens*	Lamiaceae	-	+	-
36	*Hyptis lanceolata*	Lamiaceae	+	+	-
37	*Ipomea batata*	Convolvulaceae	+	-	-
38	*Ipomea involucrata*	Convolvulaceae	+	-	-
39	*Justicia schimperi*	Acanthaceae	+	-	-
40	*Melochiacor chorifolia*	Malvaceae	+	-	-
41	*Mimosa pigra*	Fabaceae	+	-	-
42	*Mimosa pudica*	Fabaceae	+	-	-
43	*Mitracarpus villosus*	Rubiaceae	+	-	-
44	*Momordica charantia*	Cucurbitaceae	+	+	-
45	*Mucuna sloanei*	Fabaceae	+	-	-
46	*Newbouldia laevis*	Bignoniaceae	+	-	-
47	*Ocimum basilicum*	Lamiaceae	+	-	-

48	*Ocimum gratissimum*	Lamiaceae	+	-	-
49	*Oryza barthii*	Poaceae	-	+	-
50	*Panicum baumanii*	Poaceae	+	-	-
51	*Parinari curatellifolia*	Chrysobalanaceae	+	-	-
52	*Pavetta crassipes*	Rubiaceae	+	-	-
53	*Pericopsis laxiflora*	Fabaceae	+	-	-
54	*Piliostigma thonningii*	Fabaceae	+	-	-
55	*Phyllanthus floribundus*	Euphorbiaceae	+	-	-
56	*Phyllanthus muellerianus*	Euphorbiaceae	+	-	+
57	*Rotthoellia cochinchinensis*	Poaceae	+	-	-
58	*Sesamum indicum*	Pedaliaceae	+	-	-
59	*Senna obtussifolia*	Fabaceae	+	-	-
60	*Senna occidentalis*	Fabaceae	+	-	-
61	*Sida acuta*	Malvaceae	+	+	-
62	*Sida cordifolia*	Malvaceae	+	-	-
63	*Sorghum bicolor*	Poaceae	+	-	-
64	*Striga hermonthica*	Orobanchaceae	+	-	-
65	*Synedrella nodiflora*	Asteraceae	+	-	-
66	*Talinum triangulare*	Portulacaeae	+	-	-
67	*Tephrosia linearis*	Fabaceae	+	-	-
68	*Tridax procumbens*	Asteraceae	+	-	+
69	*Urena lobata*	Malvaceae	-	-	+
70	*Uvaria chamae*	Annonaceae	+	-	-
71	*Vernonia perrottetii*	Asteraceae	+	-	-

72	*Zingiber officinales*	Zingiberaceae	+	-	-

Key: + means present, - means absent
